# High Prevalence of Lipid Transfer Protein Sensitization in Apple Allergic Patients with Systemic Symptoms

**DOI:** 10.1371/journal.pone.0107304

**Published:** 2014-09-11

**Authors:** Francisca Gomez, Ana Aranda, Paloma Campo, Araceli Diaz-Perales, Natalia Blanca-Lopez, James Perkins, Maria Garrido, Miguel Blanca, Cristobalina Mayorga, Maria José Torres

**Affiliations:** 1 Allergy Service, IBIMA, Regional University Hospital of Malaga, UMA, Malaga, Spain; 2 Research Laboratory, IBIMA, Regional University Hospital of Malaga, UMA, Malaga, Spain; 3 Biotechnology Department, Center for Plant Biotechnology and Genomics, Pozuelo de Alarcon, Madrid, Spain; 4 Allergy Service, Infanta Leonor Hospital, Madrid, Spain; Cincinnati Children's Hospital Medical Center, University of Cincinnati College of Medicine, United States of America

## Abstract

**Background:**

Apple allergy manifests as two main groups of clinical entities reflecting different patterns of allergen sensitization: oral allergy syndrome (OAS) and generalized symptoms (GS).

**Objective:**

We analysed the sensitization profile to a wide panel of different components of food allergens (rMal d 1, Mal d 2, rMal d 3, rMal d 4, rPru p 3, rBet v 1 and Pho d 2) for a population of Mediterranean patients with OAS and GS to apple.

**Methods:**

Patients (N = 81) with a history of apple allergy that could be confirmed by positive prick-prick test and/or double-blind-placebo-controlled food challenge (DBPCFC), were included. Skin prick test (SPT) and ELISA were performed using a panel of inhalant, fruit and nut allergens. ELISA and ELISA inhibition studies were performed in order to analyse the sensitization patterns.

**Results:**

Thirty-five cases (43.2%) had OAS and 46 (56.8%) GS. SPT showed a significantly higher number of positive results with peach, cherry and hazelnut in those with GS. ELISA showed a significantly high percentage of positive cases to rMal d 3, rMal d 4, rPru p 3 and Pho d 2 in patients with OAS and GS compared to controls, and to rBet v 1 in patients with OAS vs controls and between OAS and GS patients. Three different patterns of recognition were detected: positive to LTP (rMal d 3 or rPru p 3), positive to profilin (rMal d 4 and Pho d 2), or positive to both. There were also patients with rMal d 1 recognition who showed cross-reactivity to rBet v 1.

**Conclusion:**

In an apple allergy population with a high incidence of pollinosis different patterns of sensitization may occur. LTP is most often involved in those with GS. Profilin, though more prevalent in patients with OAS, has been shown to sensitise patients with both types of symptoms.

## Introduction

In adults and older children Rosacea fruits are the plant foods most often responsible for allergic reactions [1]–[3]. Of these, apple allergy is a prevalent entity in Central and Northern Europe and the sensitization pattern has been studied in detail [4], [5]. Although also important in Southern Europe, it is less frequent and has thus received less attention [1], [6], [7]. Four apple allergens have been identified so far: Mal d 1, a pathogenesis-related protein (PR-10) [8]; Mal d 2, a thaumatin-like protein (TLP) [9]; Mal d 3, a lipid transfer protein (LTP) [10]; and Mal d 4, a profilin [11].

Two major groups of entities have been described according to the patterns of allergen sensitization [1], [4]–[7]: oral allergy syndrome (OAS) and generalized symptoms (GS) that include urticaria and anaphylaxis. In Central and Northern Europe, apple sensitization has been associated with initial sensitization to an aeroallergen, with Betula pollen allergen the most often involved [4], [5], [7]. Patients sensitized to Bet v 1 also respond to Mal d 1 because of the structural homology [4], [5], [7]. In fact, over 60% of Betula sensitized patients have mild symptoms after apple ingestion, usually OAS [4], [5], [7]. By contrast, in Southern European countries, apple allergic patients more frequently have GS, no association with Betula sensitization and cross-reactions to other fruits, especially peach [1], [6], [7], [12]. In this situation peach LTP, Pru p 3, is the strongest candidate for these sensitizations [1], [6], [7], [12]. Moreover, atopic patients allergic to peach and apple may have OAS in an environment where the prevalence of Betula pollen sensitization is low. In this situation, profilin is an important sensitizer [13]–[15].

As mentioned earlier, in the Mediterranean area, the origin of apple allergy is generally attributed to LTP sensitization [1], [6], [7], [12], although the relevance of sensitization to other allergens has not yet been fully established, particularly for patients with pollen allergy. Therefore OAS can be caused both by labile (such as PR-10 and profilins), as well as stable allergens (such as LTPs), whereas systemic symptoms are associated with the latter and seldom with labile allergens. The aim of this study was to undertake a detailed analysis of allergen sensitization profiles in an important number of patients diagnosed as allergic to apple from a Mediterranean population. A clinical questionnaire was used to record the allergic response or tolerance to other fruits and vegetables. Inhibition ELISA was performed using the four apple allergens identified, as well as other important related allergens such as rBet v 1, rPru p 3 and Pho d 2 (equivalent to rMal d 1, rMal d 3 and rMal d 4, respectively).

## Materials and Methods

### Patients and controls

Patients referred to the Allergy Unit of Malaga Hospital (south of Spain area) with a history of apple allergy that could be confirmed by positive prick-prick test and/or double-blind-placebo-controlled food challenge (DBPCFC), were included. Patients were classified according to symptoms: OAS when they were restricted to the skin or mucosal sites of direct contact with the allergen (itching of the oral mucosa and lips with or without angioedema immediately after eating apple), and GS when reactions involved organs far from the site of initial contact with the food (urticaria, with or without angioedema, and anaphylaxis) accompanied or not to OAS. A control group, from the same geographical area, comprised 25 non-allergic subjects with tolerance to apple. All participants completed a written informed consent and the ethical committee of our institution approved the study (CEI Provincial of Malaga).

### Diagnostic work-up

The allergological evaluation included an examination of the patient's clinical history, a detailed questionnaire, skin test, specific IgE determination and DBPCFC.

The skin test was performed according to European guidelines [16] using Golden Delicious apples for the prick-prick technique with peel and pulp tested separately, and by SPT using commercialized extract from *Phleum*, *Olea*, *Betula*, *Platanus*, *Cupressus*, *Parietaria* and *Artemisia* pollen and apple, peach, cherry and hazelnut from ALK-Abelló (Madrid, Spain). The SPT response was considered positive if the diameter of the wheal area was 3 mm greater than that induced by the negative control.

Specific IgE antibodies to apple were measured by ImmunoCAP following the manufactureŕs recommendations (Phadia, Uppsala, Sweden). A positive result was defined as a value >0.35 kUA/l.

DBPCFC was performed as described [17], except in cases with a recent history (<1 year) of anaphylaxis after apple ingestion and SPT positive to apple. Briefly, meals containing 5, 40, and 120 g of fresh ground apple with a mixture of yogurt, orange juice, coffee dried, and oatmeal flakes were freshly prepared 5 minutes before administration to prevent the loss of allergenicity. Placebo meals consisted of the same ingredients, without fresh apple. If cutaneous and/or respiratory symptoms or alterations in vital signs appeared, the procedure was stopped and the symptoms were evaluated and treated.

### Purification and quality controls

The LTPs was purified following the method previous published by Diaz-Perales [18]. Briefly, LTP was purified from defatted peel peach or apple fruit by RP-HPLC on a Vydac-C4 column (22×250 mm; particle size 10 mm; The Separations Group, Hesperia, CA, USA), followed by RP-HPLC Nucleosil 300-C4 column (8×250 mm; particle size 5 mm; Sugelabor, Madrid, Spain). Finally, proteins were analyzed by mass spectrometry, SDS-PAGE and immunoblotting.

Mal d 2 was purified from apple fruit by cation-exchange chromatography on a Bio-ScaleTM Mini Macro-Preps High S column (BioRad, Hercules,CA, USA). Following this, its purity and reactivity was analyzed by mass spectrometry, SDS-PAGE and immunoblotting.

rMal d 1 and Pho d 2 was obtained from Biomay (Vienna, Austria) and ALK-Abello (Madrid, Spain), respectively.

rMal d 4 was prepared using the protocol described by Scheurer S [19]. Briefly, the protein was cloned by polymerase chain reaction and produced in *Escherichia coli* BL21. The profilin was purified as non-fusion proteins by affinity chromatography on poly-(L-proline)-Sepharose and its purity and IgE reactivity was analyzed by SDS-PAGE and immunoblotting.

rBet v 1 was produced in *Escherichia coli* BL21 (DE3). This protein was purified under denaturing conditions from inclusion bodies and subsequently subjected to Immobilized Metal Affinity Chromatography (IMAC) followed by ion-exchange chromatography (IEC). Finally, His-Tagged rBet v 1 was analyzed by SDS PAGE and Immunoblotting using an anti-His-Tag antibody, anti-Bet v 1 monoclonal antibody and sera from patients with IgE-reactivity to Bet v 1.

### Specific IgE and inhibition studies by ELISA

ELISA assays were performed as described [18]. Costar plates (Corning, NY, USA) were coated with 5 µg/mL of rMal d 1 (Biomay, Vienna, Austria), Mal d 2, rMal d 3 and rPru p 3 (Dra Díaz-Perales, Polytechnical University of Madrid), rMal d 4 and rBet v 1 (Dr Vieths, Paul-Ehrlich Institut) and Pho d 2 (ALK-Abelló). After, the plates were blocked with blocking solution (Sigma, St. Louis,USA) for 1 h. Individual serum from patients and controls at 1∶5 dilution was added to each well. We used two negative controls, a pool of sera from non-allergic subjects and blocking buffer, and as positive controls we used patients sera with known specific IgE to proteins. The assay was completed with rabbit anti-human IgE antibody (1∶3000, DAKO, Denmark) conjugated to horseradish peroxidase (HRP). IgE binding was detected using o-phenylenediamine (OPD, DAKO). Results are expressed as absorbance units, measured at 490 nm. They were considered positive when >0.18 (mean OD + 3xSD to blocking).

For ELISA inhibition, sera were pre-incubated with different inhibitors (rMal d 3, rMal d 4, rPru p 3, Pho d 2) at final concentrations of 20, 2, and 0.2 µg/mL or with phosphate buffer for the non-inhibited serum for 3 h at room temperature. Subsequently, the inhibitor mixtures were added to plates coated with rMal d 3, rMal d 4, rPru p 3 or Pho d 2, followed by the ELISA protocol described above.

To analyse the sensitization of patients positive to rMal d 1 and rBet v 1, sera from specific IgE positive patients were pre-incubated with rMal d 1, Mal d 2, rMal d 3, rMal d 4 and rBet v 1 and added in solid phases with rMal d 1 and rBet v 1.

Percentage inhibition of IgE binding was calculated as follows: % Inhibition  =  (serum not inhibited-serum inhibited/serum not inhibited) x100.

### Statistical studies

Quantitative variables are shown as medians and interquartile ranges (IR), while qualitative variables are shown as frequencies. Medians between groups were compared using Mann-Whitney or Kruskal-Wallis tests, while χ^2^ was used to compare proportions. Differences with a p<0.05 were considered significant.

## Results

### Patient characteristics

The study included 81 patients with a diagnosis of hypersensitivity to apple. Their median age was 31 (IR:25–38) years and 62 (76.44%) were female. Thirty-five patients (43.20%) had OAS and 46 (56.79%) had GS (22 anaphylaxis (47.82%) and 24 (52.12%) urticaria). Sixty-eight cases (83.95%) showed positive prick by prick skin tests with apple peel and pulp and 55 (67.90%) positive skin test with the commercial extract for 77.14% of cases tested using prick by prick and for 57.14% with skin test. In GS patients we found that 76.08% showed skin test positivity with commercial extract and 89.13% with prick by prick. Moreover, we did not find any patient who was positive with commercial extracts and negative with prick by prick. In addition, 65 (80.24%) had serum specific IgE to apple and 62 a positive DBPCFC to apple. DBPCFC was not performed in 19 cases who had recent apple-related anaphylaxis (<1 year) and a positive skin test to apple.

Seventy-one (87.65%) were sensitized to pollen and from these 54 (76.05%) had clinical symptoms consisting of rhinitis and/or asthma; 77 (95.06%) presented symptoms with peach and 31 (38.27%) with hazelnut. The median age at symptom onset was 15 (IR:10–25) years to peach, 17 (IR:11–27) to apple, 17.5 (IR:7.75–25) to pollen and 18 (IR:13–25) to hazelnut.

Comparisons between patients with OAS and GS (Table 1) showed that although the percentage of cases with a positive SPT and specific IgE to apple was higher in those with GS, there were no significant differences between the groups in any of the variables evaluated.

**Table 1:** Comparison of demographic and clinical characteristics of the patients evaluated with OAS and GS. Statistical significance is indicated (P).

CHARACTERISTICS	OAS N = 35	GS N = 46	P
**Age in years (median, IR)**	30 (24–38)	32 (25–39)	0.583
**Sex (N, % female)**	25 (71.42)	37 (80.43)	0.430
**Apple prick by prick positive (N, %)**	27 (77.14)	41 (89.13)	0.145
**Apple commercial skin test positive (N, %)**	20 (57.14)	35 (76.08)	0.094
**Specific IgE to apple (N,%)**	27 (77.14)	38 (82.60)	0.072
**Pollen sensitization (N, %)**	31 (88.57)	40 (86.95)	0.553
**Pollen allergy (N, %)**	26 (74.28)	28 (60.86)	0.151
**Foods involved (N, %)**			0.399
Peach	32 (91.42)	45 (97.82)	
Hazelnut	13 (37.14)	17 (36.95)	
**Age at symptom onset in years (median, IR)**			
Pollen	18 (5.75–32.25)	15 (8.25–24.25	0.690
Apple	19 (12.75–29.52)	15 (11–26)	0.252
Peach	15 (11–26)	14 (9.25–24.52)	0.383
Hazelnut	23 (16–31)	15 (12.25–24.75)	0.151

Comparisons of clinical symptoms between patients with (N = 54, 66.66%) and without pollen allergy (N = 27, 33.33%) showed that in those with pollen allergy 24 (44.44%) developed GS and 30 (55.55%) OAS, whereas in those without pollen allergy, 18 (66.66%) developed GS and 9 (33.33%) OAS (Data not shown).

### Skin prick test results

Analysis of the SPT showed that 49 (60.49%) cases were positive to *Phleum*, 54 (66.67%) to *Olea*, 30 (65.22%) to *Betula*, 44 (54.32%) to *Platanus*, 41 (50.62%) to *Cupressus*, 38 (46.91%) to *Parietaria*, 35 (43.21%) to *Artemisa*, 46 (56.79%) to peach, 34 (41.97%) to cherry and 32 (39.51%) to hazelnut. Comparison between patients with OAS and those with GS showed a significantly higher percentage of positive results only with peach, cherry and hazelnut in those with GS (Table 2).

**Table 2:** Skin test results to inhalant and food allergens in patients with OAS and GS. Statistical significance is indicated (P).

ALLERGEN SOURCE	OAS N (%)	GS N (%)	P
***Phleum*** ** pollen**	24 (68.57)	25 (54.34)	0.340
***Olea*** ** pollen**	27 (77.14)	27 (58.69)	0.204
***Betula*** ** pollen**	16 (45.71)	14 (30.43)	0.220
***Platanus*** ** pollen**	17 (48.57)	27 (58.69)	0.476
***Cupressus*** ** pollen**	20 (57.14)	21 (45.62)	0.474
***Parietaria*** ** pollen**	15 (42.85)	23 (50)	0.636
***Artemisia*** ** pollen**	14 (40)	21 (45.62)	0.811
**Peach**	13 (37.14)	33 (71.73)	**0.004**
**Cherry**	10 (28.57)	24 (52.17)	**0.05**
**Hazelnut**	8 (22.85)	24 (52.17)	**0.016**

### Specific IgE results by ELISA

**Figure 1:**
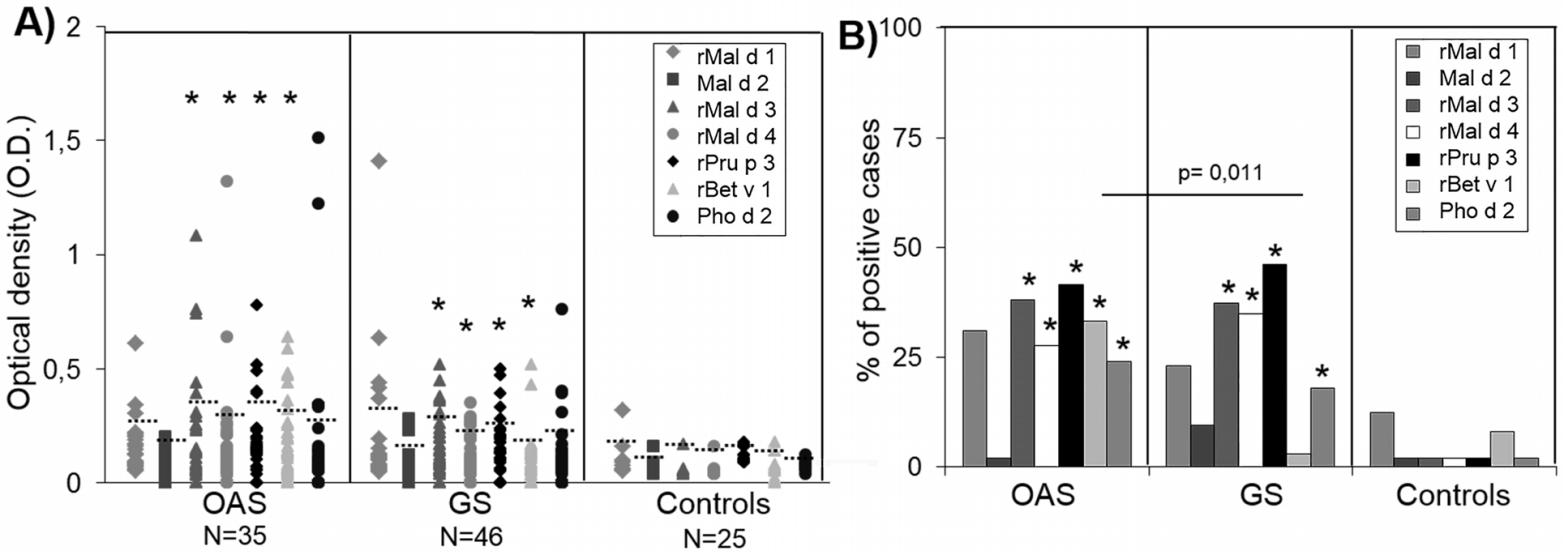
ELISA results to allergens (Mal d 1, Mal d 2, rMal d 3, rMal d 4, rPru p 3, rBet v 1 and Pho d 2) in sera from patients with OAS (N = 35) and GS (N = 46) and a control group with tolerance to apple (N = 25). (**A**) Results are expressed as individual O.D. values and median represented as a dotted line. (**B**) Results are expressed as the percentage of positive cases. (*p<0.05) represents a significant difference compared to healthy controls.

Comparisons of specific IgE determinations (Figure 1A) in OAS, GS, and controls showed significant differences between the three groups for rMal d 3 (p<0.001), rMal d 4 (p<0.001), rPru p 3 (p = 0.015) and rBet v 1 (p = 0.02). In more detail, there was an increase in levels of IgE to four allergens in OAS when compared to controls (rMal d 3: p<0.001, rMal d 4: p<0.001, rPru p 3: p = 0.007, and rBet v 1: 0.010) and in GS compared to controls (rMal d 3: p<0.001, rMal d 4: p = 0.001, rPru p 3: p = 0.013, and rBet v 1: p = 0.041), with no differences between OAS and GS. Comparisons in terms of positive percentage (Figure 1B) showed significant differences between OAS and controls for rMal d 3 (p = 0.036), rMal d 4 (p = 0.042), rPru p 3 (p = 0.009), rBet v 1 (p = 0.038) and Pho d 2 (p = 0.021) and between GS and controls for rMal d 3 (p = 0.005), rMal d 4 (p = 0.009), rPru p 3 (p = 0.007) and Pho d 2 (p = 0.045). Moreover, rBet v 1 was higher in OAS compared to GS (p = 0.011).

### Sensitization frequencies to apple allergens

We detected that 27% of the patients recognized Mal d 1, 5% to Mal d 2, 37% to Mal d 3 and 30% to Mal d 4. When we analyzed the sensitization percentages taking into account symptoms we observed no significant differences between OAS and GS. In patients with OAS, 31% of them had specific IgE to Mal d 1, 2% to Mal d 2, 38% to Mal d 3 and 28% to Mal d 4. In patients with GS, 23% had specific IgE to Mal d 1, 9% to Mal d 2, 37.50% to Mal d 3 and 35% to Mal d 4.

Based on the IgE response to allergens from LTP and profillin families, the patients were classified in three groups (Table 3): Group 1 (LTP pattern), positive to rMal d 3 and/or rPru p 3; Group 2 (Profilin pattern), positive to rMal d 4 and/or Pho d 2; and Group 3 (LTP-Profilin pattern), positive to both. We found a significant increase in the percentage of patients positive to LTP-profilin in the group with GS. Moreover, if we consider all those with an LTP response, regardless of the profilin results, there were 11 (31.42%) positive cases in the OAS group and 27 (58.69%) in the GS (p = 0.024). On the other hand, considering all those who were positive to profilin, regardless of the LTP results, 9 (25.71%) were positive in the OAS group and 18 (39.13%) in the GS group (p = 0.240).

**Table 3:** Percentages of ELISA positive cases combining allergens from LTP and profilin family in patients allergic to apple with OAS or GS.

GROUPS	PATTERN SENSITIZATION	OAS	GENERALIZED	P
**1**	**LTP**	10 (28.57%)	16 (34.78%)	0.635
**2**	**PROFILIN**	8 (22.85%)	7 (15.21%)	0.402
**3**	**LTP-PROFILIN**	1 (2.85%)	11 (23.91%)	**0.010**
**4**	**Negative**	16 (45.71%)	12 (26.08%)	0.098

### IgE recognition pattern to different allergens

**Figure 2:**
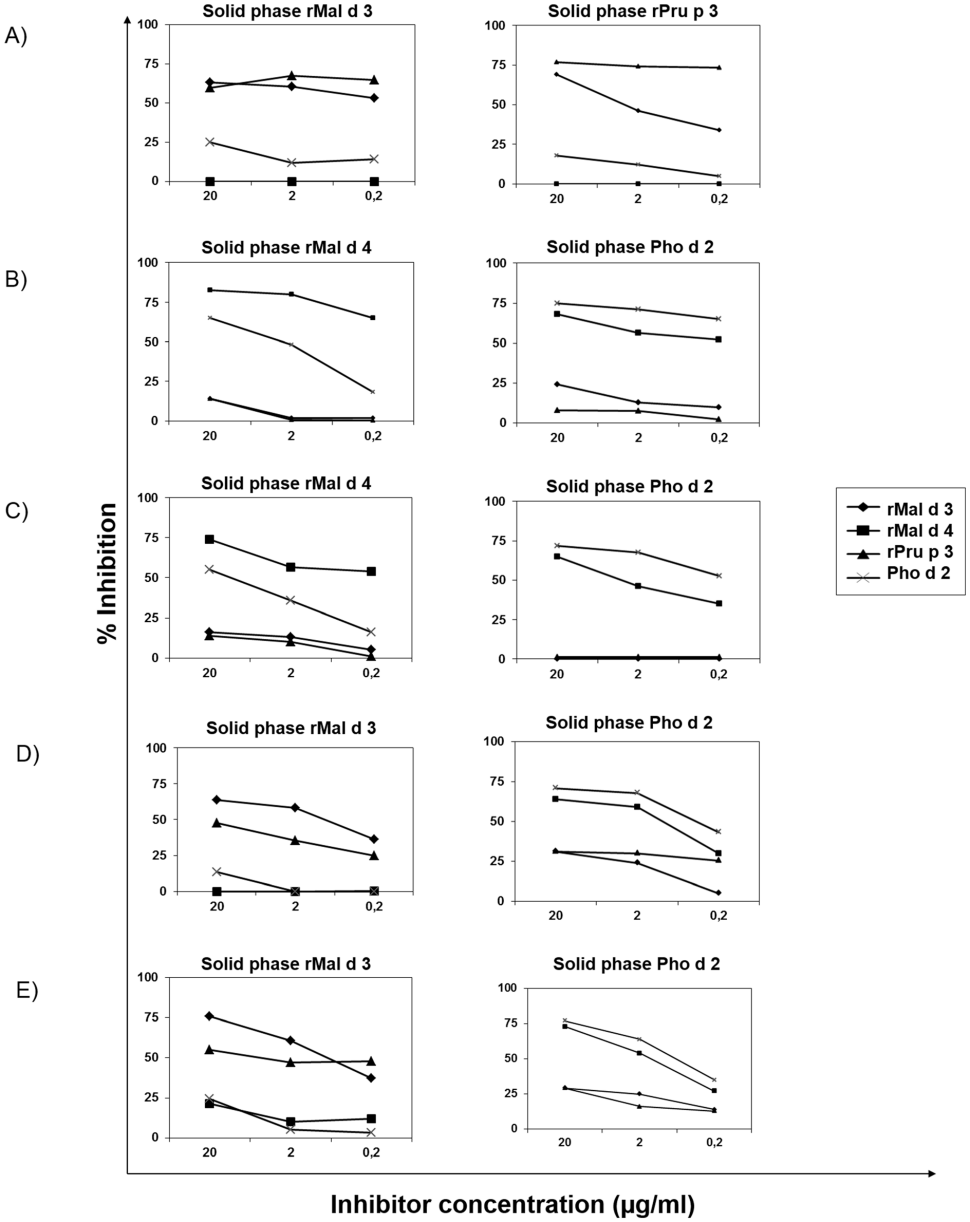
ELISA Inhibition results using rMal d 3 and rPru p 3 in the solid phase for a representative case from Group 1 with GS (A); Mal d 4 and Pho d 2 in a representative case from Group 2 with OAS (B) and a case with GS (C); and Mal d 3 and Pho d 2 in a representative case from Group 3 with OAS (D) and a case with GS (E). Sera were preincubated with decreasing concentrations (20–0.2 µg/ml) of rMal d 3, rMal d 4, rPru p 3, and Pho d 2, before they were added to the solid phase. Results are expressed as percentage inhibition.

ELISA inhibition was performed for all cases showing a sIgE level greater than 0.5 of O.D. Figure 2 shows the ELISA inhibition pattern for representative cases from each group of patients. Results from the Group 1, patients with GS, showed that using either rMal d 3 or rPru p 3 in the solid phase the strongest inhibitor was rPru p 3, followed by rMal d 3, with no inhibition by the other allergens (Figure 2A).

In Group 2, patients with either OAS or GS, using rMal d 4 in the solid phase, the highest inhibition was found with itself followed by Pho d 2; similar results were seen using Pho d 2 in the solid phase (Figure 2B and C).

Figures 2D and 2E show sera from Group 3 for patients with OAS or GS, respectively. In both cases, when using rMal d 3 in the solid phase the highest inhibition was found with rMal d 3 followed by rPru p 3. Using Pho d 2 in the solid phase we detected the highest inhibition with itself and rMal d 4.

**Figure 3:**
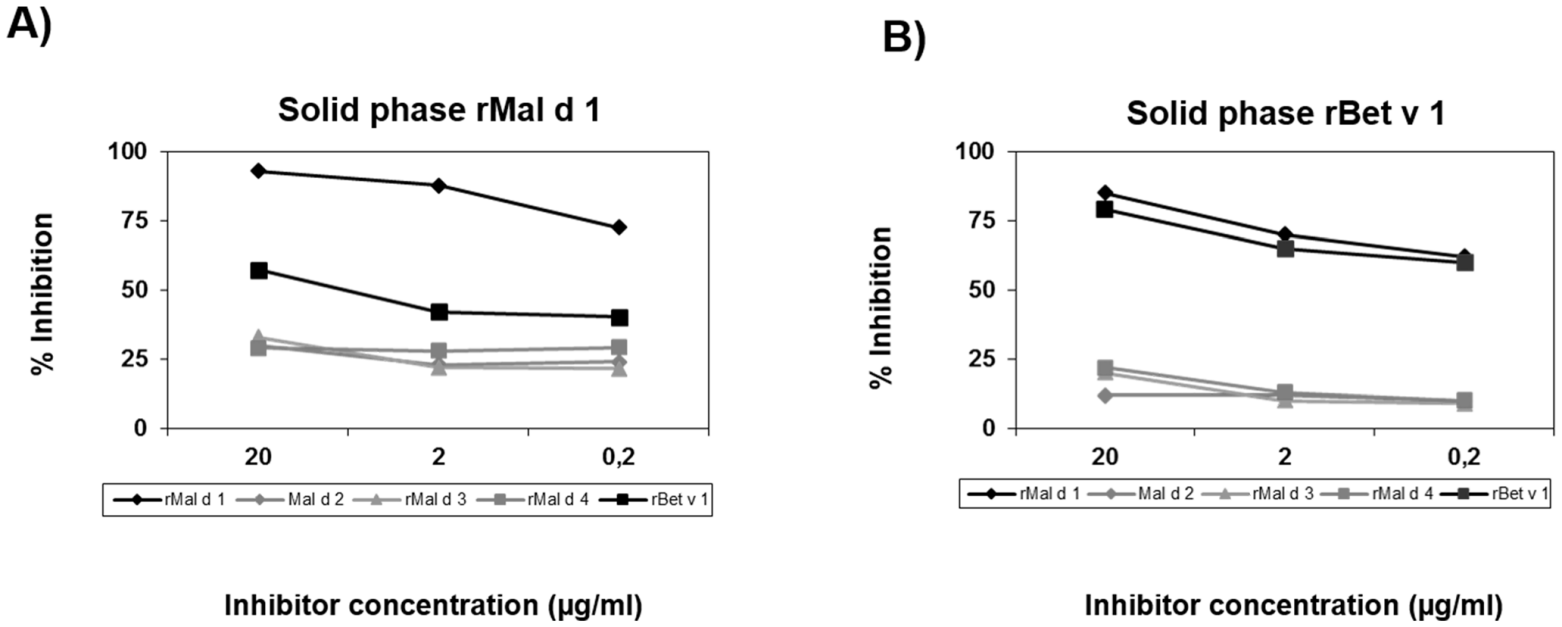
ELISA Inhibition results using in the solid phase rMal d 1 (A) and rBet v 1 (B) with different concentrations (20–0.2 µg/ml) of rMal d 1, Mal d 2, rMal d 3, rMal d 4 and rBet v 1 in a representative OAS patient with high recognition of Betv1.

In order to further analyse sensitization to Bet v 1 observed in the patients allergic to apple, ELISA inhibition studies were performed by analyzing the IgE recognition to the PR-10 proteins, rMal d 1 and rBet v 1 (Figure 3). Data showed that rMal d 1 was the most potent inhibitor regardless of the solid phase used (rMal d 1 or rBet v 1). However, rBet v 1 only showed high inhibition when the same allergen was used in the solid phase. Moreover, no inhibition was detected with Mal d 2, rMal d 3 or rMal d 4.

## Discussion

In this study we compared the sensitization pattern and IgE recognition for a wide panel of relevant purified proteins from inhalant and fruit allergens in a population of Mediterranean patients allergic to apple. Patients were selected based on a consistent clinical history, skin test, specific IgE and/or DBPCFC. Considering the clinical symptoms, in our population over 50% of patients developed GS, with almost half developing anaphylaxis. This is contrary to what occurs in Central and Northern European countries, where OAS is the most frequent clinical entity [2], [3], [5], [7]. This case series differs from a previously published study of a Spanish population, which reported a lower percentage of patients with GS (35%) [7].

For patients with GS we found that 58.69% of cases had IgE antibodies to LTP (rMal d 3 or rPru p 3), independently of profilin sensitization; thus confirming previous data showing that proteins from the LTP family are the most relevant sensitizers in apple allergy in southern Europe [6], [7], . Moreover, these patients had higher sensitization to peach, cherry and hazelnut, where LTPs are also relevant allergens [18], [22], therefore confirming that in apple allergic patients, LTP sensitization is related with more severe symptoms [7].

Considering profilin sensitization (rMal d 4 and Pho d 2), we found that 38.06% of cases were positive to these allergens only, with no differences between patients with OAS and GS (22.85% and 15.21% of cases respectively). These results indicate that when all patients with apple allergy are analysed together the picture is more complex than previously thought [6], [7], and that in some cases profilin may be the sole agent responsible for the symptoms. Nevertheless, our results are in agreement with some studies detecting a high association between profilin sensitization and OAS in peach and/or apple allergic patients [13], [14], [23].

Importantly, this study confirmed different patterns of IgE recognition through the use of detailed inhibition studies. Our data indicate the recognition of Mal d 3 with high cross-reactivity with Pru p 3 in those with LTP sensitization and of Mal d 4 with high cross-reactivity with Pho d 2 in those sensitized to profilin.

Another important point is that in our population 87.65% of all cases were sensitized to pollen with 76.05% of all cases having clinical symptoms of asthma or rhinitis. When we consider apple allergy in the context of pollen sensitization we found that those positive to these inhalant allergens had less severe symptoms as have been described by Pastorello in peach allergy where patients positive to Pru p 3 were significantly less likely to develop severe symptoms when also sensitised to Pru p 1 and Pru p 4 [23]. More specifically, among those with GS, 66.7% did not have pollen allergy compared to 44.6% who did have pollen allergy.

Although *Betula* is not normally encountered in our area we detected positive SPT to *Betula* pollen and specific IgE antibodies to Bet v 1 in 43.41% and 20.29% of cases, respectively, with no differences between patients with OAS and with GS. Differences were also observed between the *Betula* major allergen Bet v 1 and the homologous Mal d 1, with higher percentages of specific IgE antibodies in patients with OAS (33.32% and 31%, respectively) compared with GS (3% and 23.14%, respectively). These figures, although lower than those detected in central and northern Europe [7], [24], are nevertheless higher than those previously detected in a Spanish population allergic to apple [7]. In our geographical area, people are not exposed to *Betula* and the clinical expression of this sensitization is not relevant. Other, *Betula* related species, such as such as hazel and oak from the Fagales order, have been shown to cause cross-reactivity in *Betula*-free areas [25], [26]. However, in our study the inhibition results indicate that *Betula* recognition in our population could be due to cross-reactivity with Mal d 1, the latter being the primary sensitizer.

Finally, we detected a low frequency of Mal d 2 sensitization, 2% in those with OAS and 9% in those with GS. These results are similar to those detected throughout Europe [7], [27], [28].

Summarizing, we have analysed a population of patients with apple allergy and a high percentage of pollinosis. Within this population we have seen various patterns of sensitization, with LTP being the most prevalent sensitizer for patients with GS while profilin, though more prevalent for those with OAS, is an important sensitizer for patients with both type of symptoms. Moreover, Mal d 1 was also detected and associated with mild symptoms. All these results indicate that, given the different recognition patterns, it would be desirable to include profillin, LTP and also PR-10 allergens in routine diagnosis of apple allergic patients.
